# Identification and ecology of alternative insect vectors of ‘*Candidatus* Phytoplasma solani’ to grapevine

**DOI:** 10.1038/s41598-019-56076-9

**Published:** 2019-12-20

**Authors:** Fabio Quaglino, Francesco Sanna, Abdelhameed Moussa, Monica Faccincani, Alessandro Passera, Paola Casati, Piero Attilio Bianco, Nicola Mori

**Affiliations:** 1 Dipartimento di Scienze Agrarie e Ambientali - Produzione, Territorio, Agroenergia, Università degli Studi di Milano, via Celoria 2, 20133 Milano, Italy; 2 Dipartimento di Agronomia Animali Alimenti Risorse Naturali e Ambiente, Università degli Studi di Padova, Agripolis - viale dell’università, 16 – Legnaro, Padova, Italy; 3 Consorzio per la tutela del Franciacorta - via G. Verdi 53, 25030 Erbusco, BS Italy

**Keywords:** Pathogens, Plant sciences

## Abstract

Bois noir, a disease of the grapevine yellows complex, is associated with ‘*Candidatus* Phytoplasma solani’ and transmitted to grapevines in open fields by the cixiids *Hyalesthes obsoletus* and *Reptalus panzeri*. In vine-growing areas where the population density of these vectors is low within the vineyard, the occurrence of bois noir implies the existence of alternative vectors. The aim of this study was to identify alternative vectors through screening of the Auchenorrhyncha community, phytoplasma typing by *stamp* gene sequence analyses, and transmission trials. During field activities, conducted in Northern Italy in a vineyard where the bois noir incidence was extremely high, nine potential alternative insect vectors were identified according to high abundance in the vineyard agro-ecosystem, high infection rate, and harbouring phytoplasma strains characterized by *stamp* gene sequence variants found also in symptomatic grapevines. Transmission trials coupled with molecular analyses showed that at least eight species (*Aphrodes makarovi*, *Dicranotropis hamata*, *Dictyophara europaea*, *Euscelis incisus*, *Euscelidius variegatus*, *Laodelphax striatella*, *Philaenus spumarius*, and *Psammotettix alienus/confinis*) are alternative vectors of ‘*Candidatus* Phytoplasma solani’ to grapevines. These novel findings highlight that bois noir epidemiology in vineyard agro-ecosystems is more complex than previously known, opening up new perspectives in the disease management.

## Introduction

Bois noir (BN), a disease of the grapevine yellows (GY) complex, causes serious crop losses in wine-making grape varieties in the Euro-Mediterranean area and in other vine-growing countries. BN is associated with strains of ‘*Candidatus* Phytoplasma solani’ (CaPsol) (subgroup 16SrXII-A), a phloem-limited cell wall-less bacterium of the Mollicutes class^1–5^. *Hyalesthes obsoletus* Signoret (Hemiptera, Cixiidae), a polyphagous insect feeding mainly on bindweed (*Convolvulus arvensis* L.), nettle (*Urtica dioica* L.), chaste tree (*Vitex agnus-castus* L.), and stinking hawk’s beard (*Crepis foetida* L.), is the principal vector of CaPsol strains to grapevine^6–8^. In the last years, the spreading of CaPsol in vineyards where *H. obsoletus* was absent suggested the existence of additional vectors. Recently, a study conducted in Serbia demonstrated the capability of *Reptalus panzeri* Löw to transmit CaPsol to grapevine^9^. On the other hand, *Macrosteles quadripunculatus* (Kirschbaum), *Anaceratagallia ribauti* Ossiannilsson, and *Reptalus quinquecostatus* (Dufour) were found able to transmit CaPsol in experimental conditions but no evidence of transmission of the pathogen to grapevine is available yet^10–12^. Furthermore, numerous CaPsol-harbouring planthopper (Cixiidae) and leafhopper (Cicadellidae) species were found within or around BN-affected vineyards^13,14^. Considering such evidences, the identification of which insect species can effectively transmit the phytoplasma to grapevine is of paramount importance to formulate effective control strategies to reduce the BN incidence.

Based on *tufB* gene sequence analysis, three CaPsol *tuf*-types, identified in both BN-affected grapevines and non-crop host plants, were associated with two distinct CaPsol ecological cycles related to bindweed (*tuf*-type b) and nettle [*tuf*-type a and ab (formerly known as b2)]^6,15,16^. A study conducted in Eastern Europe reported the direct epidemiological role of chaste trees as CaPsol source in the *H. obsoletus*-mediated transmission to grapevine^7^. Moreover, in the Balkan region, it was recently highlighted that *H. obsoletus* population related to stinking hawk’s beard can acquire CaPsol *tuf*-type b from this source plant and transmit it to grapevine^8^. A larger genetic diversity among CaPsol strains was described by molecular characterization of less conserved genes (*i.e*., *secY*, *vmp1*, and *stamp*)^17,18^. Interestingly, studies focused on *stamp* gene molecular markers improved the knowledge on CaPsol strain population structure and dynamics^19^, revealing the phytoplasma transmission ways in vineyard agro-ecosystems^7,20^. In detail, it was found that CaPsol strains grouped in diverse *stamp*-based phylogenetic clusters are associated with bindweed- and nettle-related ecological cycles^15,16,21^. Moreover, recent findings highlighted that in Tuscany (central Italy) BN is prevalently associated with a CaPsol strain never found before in grapevine but detected exclusively in other host plants^22^. This reinforced the evidence of the existence of alternative BN epidemiological cycles that could include other insect vectors able to transmit CaPsol to grapevine.

Thus, in the present study, we investigated the composition of Auchenorrhyncha community in vineyards where CaPsol is significantly spreading, identified putative insect vectors and conducted transmission trials, along with molecular analyses to investigate the capability of prevalent species of Cixiidae and Cicadellidae to transmit CaPsol to grapevine.

## Results

### Auchenorrhyncha community description

During field activities, 1018 and 896 adult insects were collected in 2013 and 2014, respectively. Stereomicroscope analysis, based on observation of morphological characters, allowed the identification of 48 distinct taxonomic groups, 44 defined at species level and four at genus level, belonging to a total of nine different families (Table 1). The most represented family was Cicadellidae, as 30 out of 48 taxonomic groups belong to this family, adding up to 377 individuals out of the 1018 captured in 2013 (37%) and 377 out of the 896 captured in 2014 (42%). The other most relevant families were Delphacidae (representing 17.7% and 18.5% of insects captured in 2013 and 2014, respectively), Cixiidae (representing 11% and 13.5% of insects captured in 2013 and 2014, respectively) (around the borders), and Aphrophoridae (representing 9.1% and 10.1% of insects captured in 2013 and 2014, respectively), mostly represented by the species *Laodelphax striatella*, *H. obsoletus*, *Philaenus spumarius*, respectively. Cercopidae (most of which belong to the *Cercopis vulnerata* species), Dictyopharidae (with *Dictyophara europaea* species) and Flatidae (with *Metcalfa pruinosa* species) families were highly represented with 24% and 15% of captured insects in 2013 and 2014, respectively. The remaining two families, Caliscelidae and Membracidae, had the lowest abundance (less than 1%) among captured insects. The grapevine leafhoppers *Empoasca vitis* (Göthe) and *Zygina rhamni* Ferrari were not considered, as their population density was very low.

**Table 1:** Captures and infection rate of CaPsol potential insect vectors in the examined vineyard in 2013 and 2014.

Family	Subfamily	Insect	2013				2014			
			Number of captured specimens	Number of pools	Number of CaPsol infected pools	Proportion of infected pools	Number of captured specimens	Number of pools	Number of CaPsol infected pools	Proportion of infected pools
Cixiidae	Cixiinae	*Cixius spp*. Latreille, 1804	2	2	—	—	—	—	—	—
Cixiidae	Cixiinae	*Hyalesthes obsoletus* Sign., 1865	100	44	13	30	113	43	10	23
Cixiidae	Cixiinae	*Hyalesthes scotti* Ferrari, 1882	8	6	—	—	4	2	—	—
Cixiidae	Cixiinae	*Reptalus spp*. Emeljanov, 1971	2	2	—	—	4	4	—	—
Delphacidae	Asiracinae	*Asiraca clavicornis (*F., 1794)	6	6	2	33	—	—	—	—
Delphacidae	Delphacinae	*Dicranotropis hamata* (Boh., 1847)	31	15	2	13	12	7	1	14
Delphacidae	Delphacinae	*Laodelphax striatella* (Fall., 1826)	121	56	6	11	71	30	2	7
Delphacidae	Delphacinae	*Toya propinqua* (Fieb., 1866)	20	9	—	—	81	24	2	8
Delphacidae	Kelisiinae	*Kelisia guttulifera* (Kbm., 1868)	2	2	—	—	—	—	—	—
Delphacidae	Stenocraninae	*Stenocranus major* (Kbm., 1868)	—	—	—	—	2	2	—	—
Dictyopharidae	Dictyopharinae	*Dictyophara europaea* (L., 1767)	47	47	—	—	59	59	12	20
Flatidae	Flatinae	*Metcalfa pruinosa* (Say, 1830)	68	35	9	26	79	41	1	2
Cercopidae	Cercopinae	*Cercopis vulnerata* Rossi, 1807	120	120	2	2	—	—	—	—
Cercopidae	Cercopinae	*Haematoloma dorsatum* (Ahr., 1812)	12	7	—	—	—	—	—	—
Aphrophoridae	Aphrophorinae	*Philaenus spumarius* (L., 1758)	93	28	2	7	91	53	14	26
Membracidae	Centrotinae	*Centrotus cornutus* (L., 1758)	1	1	—	—	—	—	—	—
Membracidae	Smiliinae	*Stictocephala bisonia* Kopp & Yon, 1977	1	1	—	—	3	3	1	33
Cicadellidae	Agallinae	*Anaceratagallia ribauti* (Oss., 1938)	—	—	—	—	5	4	—	—
Cicadellidae	Aphrodinae	*Aphrodes makarovi* Zachv., 1948	29	21	5	24	2	2	—	—
Cicadellidae	Cicadellinae	*Cicadella viridis* (L., 1758)	43	35	6	17	142	123	21	17
Cicadellidae	Cicadellinae	*Evacanthus acuminatus* (F., 1794)	3	3	2	67	—	—	—	—
Cicadellidae	Deltocephalinae	*Anoplotettix fuscovenosus* (Ferr., 1882)	13	12	1	8	—	—	—	—
Cicadellidae	Deltocephalinae	*Arthaldeus striifrons* (Kbm., 1868)	2	1	—	—	2	1	—	—
Cicadellidae	Deltocephalinae	*Aconurella prolixa* (Leth., 1885)	1	1	—	—	6	3	—	—
Cicadellidae	Deltocephalinae	*Allygidius furcatus* (Ferr., 1882)	25	24	2	8	33	9	1	10
Cicadellidae	Deltocephalinae	*Euscelis incisus* (Kbm., 1858)	30	15	2	13	11	10	1	10
Cicadellidae	Deltocephalinae	*Euscelidius variegatus* (Kbm., 1858)	58	31	3	10	21	20	1	5
Cicadellidae	Deltocephalinae	*Fieberiella florii* (Stål, 1864)	5	5	1	20	—	—	—	—
Cicadellidae	Deltocephalinae	*Goniagnathus brevis* (H.-S., 1835)	5	5	—	—	—	—	—	—
Cicadellidae	Deltocephalinae	*Hishimonus hamatus* Kuoh, 1976	4	4	—	—	13	13	1	8
Cicadellidae	Deltocephalinae	*Japananus hyalinus* (Osb., 1900)	2	2	—	—	10	5	1	20
Cicadellidae	Deltocephalinae	*Jassargus flori* (Fieb., 1869)	—	—	—	—	17	9	—	—
Cicadellidae	Deltocephalinae	*Macrosteles cristatus* (Rib., 1927)	5	4	—	—	1	1	—	—
Cicadellidae	Deltocephalinae	*Mocydia crocea* (H.-S., 1837)	4	2	—	—	5	3	—	—
Cicadellidae	Deltocephalinae	*Mocydiopsis spp*. Rib. 1939	2	2	—	—	1	1	—	—
Cicadellidae	Deltocephalinae	*Neoaliturus fenestratus* (H.-S., 1834)	5	2	1	50	—	—	—	—
Cicadellidae	Deltocephalinae	*Ophiola sp*. Edwards, 1922	—	—	—	—	1	1	—	—
Cicadellidae	Deltocephalinae	*Orientus ishidae* (Mats., 1902)	—	—	—	—	8	8	6	75
Cicadellidae	Deltocephalinae	*Psammotettix alienus/confinis* (Dhlb, 1850)	30	12	—	—	81	34	4	12
Cicadellidae	Deltocephalinae	*Scaphoideus titanus* (Ball, 1932)	4	1	1	100	8	8	2	25
Cicadellidae	Deltocephalinae	*Thamnotettix zelleri* (Kbm., 1868)	5	5	1	20	9	5	1	20
Cicadellidae	Idiocerinae	*Balcanocerus larvatus* (H.-S., 1835)	5	5	—	—	—	—	—	—
Cicadellidae	Macropsinae	*Hephathus nanus* (H.-S., 1835)	70	25	—	—	1	1	—	—
Cicadellidae	Macropsinae	*Macropsis fuscula* (Zett., 1828)	13	6	—	—	—	—	—	—
Cicadellidae	Megophthalminae	*Megophthalmus scanicus* (Fall. 1806)	4	4	2	50	—	—	—	—
Cicadellidae	Typhlocybinae	*Dikraneura variata* Hardy, 1850	1	1	—	—	—	—	—	—
Cicadellidae	Typhlocybinae	*Typhlocyba quercus* (F., 1777)	9	5	—	—	—	—	—	—
Caliscelidae	Caliscelinae	*Caliscelis bonellii* (Latreille, 1807)	7	7	—	—	—	—	—	—
	Total	1018	621	63	10	896	529	82	16	

### Identification and molecular characterization of ‘*Ca*. P. solani’ in insects and grapevines

PCR-based amplification of *stamp* gene revealed the presence of CaPsol in 63 out of 621 analyzed insect pools from 2013 and 82 out of 529 pools from 2014, belonging to 19 species in 2013 and 18 species in 2014, 12 of which are common to both years (Table 1). The amplification of *stamp* gene also revealed the presence of CaPsol in 54 grapevine leaf samples (29 out of 30 in 2013, and 25 out of 30 in 2014). The 112 *stamp* fragments (StampF1/StampR1) of the expected size, amplified in 2013 and 2014 (58 from insects and 54 from grapevines) were sequenced (Table 2). Based on the nucleotide sequence identity, six *stamp* sequence variants (here named GuSt1 to GuSt6) were identified. CaPsol strains characterized by *stamp* sequence variants GuSt1, GuSt2, GuSt3 and GuSt5 were identified in both insects and grapevines, while GuSt4 and GuSt6 were found only in insects (Table 2). Comparison of such sequence variants (GuSt1 to GuSt6) with the previously published dataset^19^ revealed that they were identical to the previously described sequence variants St5 [representative strain GGY, GenBank Accession Number (Acc. No.) FN813256], St11 (representative strain 19–25, Acc. No. FN813267), St19 (representative strain CrHo13_1183, Acc. No. KJ469719), St21 (representative strain Aa21, Acc. No. KJ145380), St30 (representative strain Vv24, Acc. No. KC703022) and St36 (representative strain Carv2, Acc. No. KT184880), respectively (Table 2). In detail, in 2013 and 2014, insects contained CaPsol strains characterized by *stamp* sequence variants St5 (63.8%), St11 (3.5%), St19 (6.9%), St21 (1.7%), St30 (15.5%) and St36 (8.6%). Grapevines harboured CaPsol strains characterized by *stamp* sequence variants St5 (50%), St11 (22.2%), St19 (14.8%) and St30 (13%) (Table 2).

**Table 2:** *Stamp* sequence variants of CaPsol identified in symptomatic grapevines and potential insect vectors.

Species	Year	CaPsol infected pools	Sequenced	CaPsol strain (*stamp* gene sequence variant)					
				GuSt1 (St5)	GuSt2 (St11)	GuSt3 (St19)	GuSt4 (St21)	GuSt5 (St30)	GuSt6 (St36)
*Vitis vinifera* L., 1753	2013	29	29	15	5	4	—	5	—
	2014	25	25	12	7	4	—	2	—
*Hyalesthes obsoletus* Sign., 1865	2013	13	5	4	—	—	—	1	—
	2014	10	4	1	1	—	1	1	—
*Asiraca clavicornis* (F., 1794)	2013	2	1	1	—	—	—	—	—
*Dicranotropis hamata* (Boh., 1847)	2013	2	1	—	1	—	—	—	—
	2014	1	1	1	—	—	—	—	—
*Laodelphax striatella* (Fall., 1826)	2013	6	3	3	—	—	—	—	—
	2014	2	1	1	—	—	—	—	—
*Toya propinqua* (Fieb., 1866)	2014	2	1	1	—	—	—	—	—
*Dictyophara europaea* (L., 1767)	2014	12	5	2	—	—	—	2	1
*Metcalfa pruinosa* (Say, 1830)	2013	9	1	1	—	—	—	—	—
	2014	1	—	—	—	—	—	—	—
*Cercopis vulnerata* Rossi, 1807	2013	2	2	2	—	—	—	—	—
*Philaenus spumarius* (L., 1758)	2013	2	2	—	—	2	—	—	—
	2014	14	4	3	—	—	—	1	—
*Stictocephala bisonia* Kopp & Yon, 1977	2014	1	—	—	—	—	—	—	—
*Aphrodes makarovi* Zachv., 1948	2013	5	3	3	—	—	—	—	—
*Cicadella viridis* (L., 1758)	2013	6	2	2	—	—	—	—	—
	2014	21	5	3	—	—	—	2	—
*Evacanthus acuminatus* (F., 1794)	2013	2	1	—	—	—	—	—	1
*Allygidius furcatus* (Ferr., 1882)	2013	2	1	1	—	—	—	—	—
	2014	1	1	—	—	—	—	—	1
*Anoplotettix fuscovenosus* (Ferr., 1882)	2013	1	1	—	—	—	—	—	1
*Euscelis incisus* (Kbm., 1858)	2013	2	1	1	—	—	—	—	—
	2014	1	1	—	—	1	—	—	—
*Euscelidius variegatus* (Kbm., 1858)	2013	3	2	2	—	—	—	—	—
	2014	1	1	1	—	—	—	—	—
*Fieberiella florii* (Stål, 1864)	2013	1	1	—	—	—	—	—	1
*Hishimonus hamatus* Kuoh, 1976	2014	1	1	—	—	—	—	1	—
*Japananus hyalinus* (Osb., 1900)	2014	1	1	1	—	—	—	—	—
*Neoaliturus fenestratus* (H.-S., 1834)	2013	1	—	—	—	—	—	—	—
*Orientus ishidae* (Mats., 1902)	2014	6	2	1	—	1	—	—	—
*Psammotettix alienus/confinis* (Dhlb, 1850)	2014	4	3	2	—	—	—	1	—
*Scaphoideus titanus* Ball, 1932	2013	1	—	—	—	—	—	—	—
	2014	2	—	—	—	—	—	—	—
*Thamnotettix zelleri* (Kbm., 1868)	2013	1	—	—	—	—	—	—	—
	2014	1	—	—	—	—	—	—	—
*Megophthalmus scanicus* (Fall. 1806)	2013	2	—	—	—	—	—	—	—

**Figure 1:**
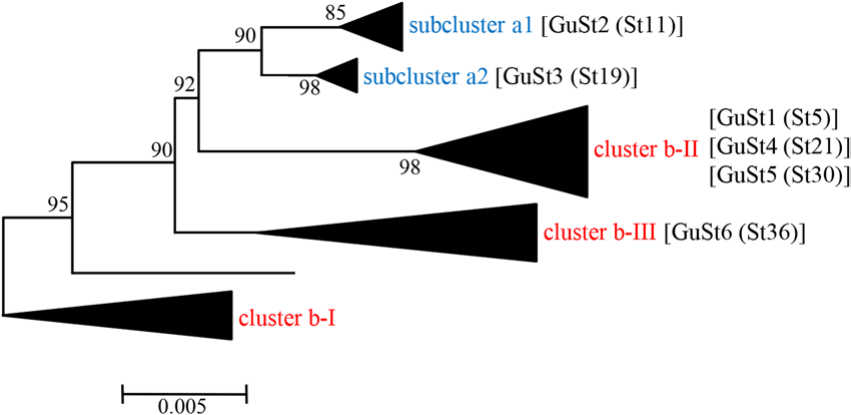
Unrooted phylogenetic tree inferred from *stamp* gene nucleotide sequences of BNp strains representative of *stamp* sequence variants identified in this study (Table 2) and previously described^19^. The evolutionary history was inferred using the Neighbor-Joining method. The optimal tree with the sum of branch length = 0.33585582 is shown. The percentage of replicate trees in which the associated taxa clustered together in the bootstrap test (1000 replicates) are shown next to the branches. The tree is drawn to scale, with branch lengths in the same units as those of the evolutionary distances used to infer the phylogenetic tree. The evolutionary distances were computed using the Jukes-Cantor method and are in the units of the number of base substitutions per site. The analysis involved 64 nucleotide sequences. All ambiguous positions were removed for each sequence pair. There were a total of 495 positions in the final dataset. Evolutionary analyses were conducted in MEGA6. Details on the distribution of CaPsol *stamp* sequence variants in BNp hosts are available in Fig. S1.

The alignment of *stamp* nucleotide sequences of CaPsol strains representative of the GuSt sequence variants identified in Gussago (GuSt1 to GuSts6) and those previously described (St1 to St58)^19^ was used for generating a phylogenetic tree in which three main clusters (b-I, b-II, b-III) and two subclusters (a1 and a2) were observed. CaPsol strains identified in the present study are found in four of these groups, as no strain clusters within the b-I cluster. CaPsol strains sharing the *stamp* sequence variant St19 (12 strains) grouped in the nettle-related subcluster a2; those with sequence variant St11 (14 strains) grouped in the nettle-related subcluster a1; those with sequence variants St5, St21 and St30 (81 strains) grouped in the bindweed-related cluster b-II; those with sequence variant St36 (five strains) in the bindweed-related cluster b-III (Fig. 1, S1).

### Identification of ‘*Ca*. P. solani’ insect vectors by transmission trials

Based on the criteria applied to select potential CaPsol insect vectors to be tested, transmission trials were conducted in 2015 and/or 2016 on the insect species *Aphrodes makarovi* Zachvatkin*, Cicadella viridis* L., *Dicranotropis hamata* Boheman, *Dictyophara europaea* Spinola, *Euscelis incisus* Kirschbaum, *Euscelidius variegatus* Kirschbaum, *Hyalesthes obsoletus*, *Laodelphax striatella*, *Philaenus spumarius* and *Psammotettix alienus/confinis* (Table 3). Due to the fact that dead specimens of both *P. alienus* and *P. confinis* were found in the cage after the transmission period, it is not possible to distinguish the species transmission ability.

**Table 3:** Identification of CaPsol insect vectors by transmission trials to grapevine and molecular analyses in 2015 and 2016. Sympt, potted grapevines with typical BN symptoms (yellowing and downwards rolling of leaves and lack of cane lignifications).

Species	Trial year	Number of insects				Number of grapevines		
		Total	Infected	Sequenced	CaPsol strain (number)	Total	Infected (CaPsol strain)	
							October (same year)	July (following year)
*Hyalesthes obsoletus* Sign., 1865	2015	15	8	8	St5 (8)	2	2 (St5)	2 (St5)
	2016	24	22	4	St5 (3); St19 (1)	2	—	1 (St5) Sympt
*Dicranotropis hamata* (Boh., 1847)	2015	120	5	3	St5 (3)	3	—	2 (St5)
	2016	4	—	—	—	1	—	1
*Laodelphax striatella* (Fall., 1826)	2015	46	—	—	—	2	—	—
	2016	16	12	6	St5 (4); St11 (1); St19 (1)	2	—	1 (St5)
*Dictyophara europaea* (L., 1767)	2016	26	25	5	St5 (5)	3	—	1 (St5)
*Philaenus spumarius* (L., 1758)	2016	20	18	3	St5 (3)	4	—	2 (St5)
*Aphrodes makarovi* Zachv., 1948	2016	5	4	2	St5 (2)	2	—	1 (St5)
*Cicadella viridis* (L., 1758)	2015	16	—	—	—	1	—	—
	2016	15	13	—	—	1	—	—
*Euscelis incisus* (Kbm., 1858)	2015	12	4	4	St5 (3); St19 (1)	3	—	3 (St5)
	2016	20	18	12	St5 (11); St19 (1)	3	—	3 (St5) Sympt
*Euscelidius variegatus* (Kbm., 1858)	2015	10	—	—	—	3	—	—
	2016	52	51	12	St5 (12)	5	1 (St5)	2 (St5)
*Psammotettix alienus/confinis* (Dhlb, 1850)	2015	100	—	—	—	3	—	—
	2016	49	43	16	St5 (14); St11 (1); St19 (1)	4	—	2 (St5)
No insect (control)	2015	—	—	—	—	3	—	—
	2016	—	—	—	—	5	—	—

In the 2015 transmission trials, *stamp* gene amplification and SYBR Green real-time amplification assay allowed the detection of CaPsol in *D. hamata*, *E. incisus* and *H. obsoletus* specimens, and in leaf samples (collected one year after the transmission trials, July 2016) of the grapevines on which each of these insect species was forced to feed. Only grapevines hosting *H. obsoletus* were found infected by CaPsol as early as October 2015, the end of the growing season in which transmission trials were conducted. No amplification was obtained from insect specimens of *C. viridis*, *E. variegatus*, *L. striatella*, or *P. alienus/confinis* nor from the leaf samples of the grapevines on which each of these insect species was forced to feed (Table 3).

In the 2016 transmission trials, *stamp* gene amplification and SYBR Green real-time amplification allowed the detection of CaPsol in *A. makarovi*, *D. europaea*, *E. incisus*, *E. variegatus*, *H. obsoletus*, *L. striatella*, *P. spumarius* and *P. alienus/confinis* specimens, and in leaf samples (collected one year after the transmission trials, July 2017) of the grapevines on which each of these insect species was forced to feed. Only one grapevine plant hosting *E. variegatus* was found to be infected by CaPsol as early as October 2016, the end of the growing season in which transmission trials were conducted. No amplification was obtained from insect specimens of *D. hamata*; despite this, the grapevine plant on which this insect was forced to feed was found to be infected. On the contrary, CaPsol was detected in *C. viridis* specimens but no amplification was obtained from the grapevine plant on which this insect was forced to feed. Furthermore, in both years no amplification was obtained from control plants, kept in controlled conditions without insects (Table 3). Moreover, *stamp* gene nucleotide sequences were obtained from 75 representative CaPsol-harbouring insect specimens forced to feed on the 24 grapevine plants that were found to be CaPsol-infected after the transmission trials. Sequence identity analysis evidenced that (i) all 24 infected grapevines harboured CaPsol strains characterized by the same *stamp* sequence variant (St5); (ii) in each of the nine insect species forced to feed on these 24 grapevines (all species used in the transmission bioassay except *C. viridis*) the prevalent CaPsol strains were characterized by the same *stamp* sequence variant (St5, 68 out of 75 specimens); CaPsol strains characterized by the *stamp* sequence variant St19 and St11 were identified in five and two out of 75 insect specimens, respectively; (iii) at least one insect specimen that was forced to feed on each of the 24 infected grapevines harboured CaPsol strain characterized by the *stamp* sequence variant St5 (Table 3). Yellowing and downwards rolling of leaves and lack of cane lignification were observed in September 2018 in one and two grapevines where *H. obsoletus* and *E. incisus* were confined, respectively.

### Plants eaten by alternative insect vectors

*rbcL* gene was amplified in 59 out of 154 insects of eight species. No amplification was obtained for *L. striatella* and *P. alienus/confinis*. BLAST analysis of *rbcL* nucleotide sequences showed that, with the exception of *A. makarovi*, grapevine was the most prevalent plant on which all the insects have had their last meal. Moreover, specimens of the species *D. hamata*, *D. europaea*, *E. incisus*, and *P. spumarius* had their last meal on nettle and/or *Crepis* spp. (Table 4).

**Table 4:** Identification of the plants present in the gut of the insect species tested in CaPsol transmission trials by *rbcL* gene amplification and sequence analysis.

Species	Number of insects		Plant in the insect gut		
	Analyzed	*rbcL* PCR-positive	Number of insects	GenBank closest relative plant (Acc. No.)	% Identity
*Hyalesthes obsoletus* Sign., 1865	23	1	1	*Vitis vinifera* (MG946878)	99
*Dicranotropis hamata* (Boh., 1847)	8	2	1	*Urtica dioica* (MG946931)	99
			1	*Vitis vinifera* (AJ419718)	97
*Laodelphax striatella* (Fall., 1826)	10	—	—	—	—
*Dictyophara europaea* (L., 1767)	10	6	3	*Vitis vinifera* (MG946878)	99
			1	*Crepis elongata* (JQ933285)	98
			1	*Urtica dioica* (MG946931)	98
			1	*Pisum sativum* (MG917089)	99
*Philaenus spumarius* (L., 1758)	16	11	7	*Vitis vinifera* (MG946878)	99
			1	*Vicia cracca* (KP699058)	98
			1	*Potentilla hebiichigo* (MG742490)	96
			1	*Urtica dioica* (MG946931)	94
			1	*Daucus pumilus* (KX832312)	98
*Aphrodes makarovi* Zachv., 1948	1	1	1	*Peltophorum pterocarpum* (AM234243)	95
*Cicadella viridis* (L., 1758)	24	8	5	*Vitis vinifera* (MG946878)	98
			1	*Crepis capillaris* (KM360738)	98
			1	*Laportea interrupta* (KM586531)	87
			1	*Pisum sativum* (MG917089)	95
*Euscelis incisus* (Kbm., 1858)	22	20	10	*Vitis vinifera* (MG946878)	99
			2	*Vicia spp*. (KP699053)	98
			1	*Potentilla spp*. (MG742490)	96
			1	*Crepis spp*. (KF602078)	93
			1	*Pisum sativum* (MG859922)	95
			1	*Corylus spp*. (MF996573)	99
			1	*Xanthoceras sorbifolium* (KP088923)	81
			1	*Ternstroemia gymnanthera* (MF179490)	96
			1	*Musa coccinea* (MH603431)	96
			1	*Nicotiana sylvestris* (KM025249)	98
*Euscelidius variegatus* (Kbm., 1858)	17	10	6	*Vitis vinifera* (MG946878)	98
			2	*Nicotiana spp*. (KU199713)	98
			1	*Berberidopsis corallina* (EU002274)	96
			1	*Quiina glaziovii* (JX664069)	78
*Psammotettix alienus/confinis* (Dhlb, 1850)	23	—	—	—	—

## Discussion

In Euro-Mediterranean regions the main insect vector of ‘*Ca*. P. solani’ (CaPsol) is *Hyalesthes obsoletus*^6,7^. Management of its host plants in the vineyards and surrounding areas is therefore considered crucial for BN control^23,24^. The existence of additional vectors has been theorized from the observations that BN incidence is not always correlated to high densities or presence of *H. obsoletus* populations^12,14,25^. Thus, several studies, conducted to discover alternative insect vectors, detected more than 35 insect species harbouring CaPsol, 16 of which were found to be able to transmit the phytoplasma^10–14,26^. Among these 16 species, only *Reptalus panzeri* and *Macrosteles quadripunctulatus* Fieber have been proven as vectors of CaPsol to grapevine plants in Serbia and Spain^9,10^. The small number of effective vectors compared to CaPsol-infected insects is related to the specific phytoplasma-vector recognition mechanism^27,28^, which involves the binding of insect cytoskeleton proteins with the antigenic membrane protein encoded by the CaPsol *stamp* gene^29^. Thus, *stamp*-based molecular typing of CaPsol strains has been employed to identify its insect vectors and transmission routes in this and previous studies^7,12^.

In order to optimize the experimental conditions allowing identification of CaPsol insect vectors, this study was conducted in a vineyard where BN incidence was extremely high without any correlation with *H. obsoletus* density and distribution, the ground cover had many CaPsol host plants, and the agro-ecosystem included a high plant biodiversity (presence of forest, grass and broadleaf species). In fact, probably due to this high biodiversity, many more insect species were found in the target area compared to a previous study conducted in Lombardy Region^30^. The differences in insect population presence and density observed in 2013 and 2014 should be explained by diverse climate conditions (dry and cold in 2013, wet and hot in 2014) and consequently by grass cover management within and around the vineyard, such as cutting and/or mowing, that affect the Auchenorrhyncha population density as previously demonstrated^24^.

Within the investigated Auchenorrhyncha community, nine potential insect vectors, besides *H. obsoletus*, were identified according to high abundance in the vineyard agro-ecosystem, high CaPsol-infection rate, and harbouring CaPsol strains characterized by *stamp* gene sequence variants undistinguishable from those found in symptomatic grapevines.

Interestingly, except *D. hamata*, an extraordinary infection rate was found in the tested insects, in agreement with data obtained in previous study conducted on other phytoplasma insect vectors^31^. The different temperatures registered in the two investigated years could also explain the diverse CaPsol-infection rates observed, as reported for *Candidatus* Phytoplasma asteris (CYP) in *Chrysanthemum carinatum* and its vector *Macrosteles quadripunctulatus*^32,33^. In this CYP epidemics the mean latency period in the insect and in the host plant was faster at high temperatures than low ones as consequence of faster phytoplasma multiplication in the host plants and higher frequency of feeding bouts of vectors at higher temperatures (Maggi *et al*., 2004).The difference in the infection rate between the two investigated years was probably due to the diverse insect collection period.

Transmission trials proved that eight insect species are vectors of CaPsol to grapevine. Among these, *Euscelidius variegatus* and *Euscelis incisus* were previously reported as able to transmit the pathogen to *in vitro* grapevine plantlets^34^ and to solanaceous plants^35^, respectively; the other six insect species (*Dicranotropis hamata, Laodelphax striatella*, *Dictyophara europaea, Philaenus spumarius, Aphrodes makarovi*, *Psammotettix alienus*/*confinis*) were found harbouring CaPsol in vineyards but there was no evidence of transmission to grapevine (Table 5). Moreover, *D. hamata* and *L. striatella* are related to the species *Javesella discolor* (Boheman 1847) (Delphacidae, Delphacinae) proved to be able to transmit CaPsol to artificial diet medium^26^; *Psammotettix alienus*/*confinis* is related to the species *Psammotettix striatus*, previously proved to be able to transmit CaPsol to artificial diet medium^36^; *A. makarovi* is related to the species *A. bicincta* proved to be able to transmit CaPsol to various plants in Europe^35^.

**Table 5:** Host plants, hosting/vectoring CaPsol, occurrence, and biology of alternative CaPsol vectors.

Species	Host plant	Survival on grapevine	Host/vector of CaPsol	Abundance, development cycle, adult presence in Northern Italian vineyards
*Dicranotropis hamata* (Boh., 1847)	Poaceae^40,41,52^	not available	host^51^	Uncommon – 2 generations/year. Overwintering as nymphal stage. Adult presence: mid-April - end of October^42,43,52^
*Laodelphax striatella* (Fall., 1826)	Poaceae, Juncaceae, Cyperaceae^40,41,52^	not available	host^36^	Common – 2 generations/year. Overwintering as egg stage. Adult presence: beginning of June - end of November^42,43,52^
*Dictyophara europaea* (L., 1767)	Polyphagous^52^	2–6 days^53^	host^53,54^	Common – 1 generation/year. Overwintering as egg stage. Adult presence: end of June - beginning of October^55^
*Philaenus spumarius* (L., 1758)	Polyphagous^40,41,52^	>10 days^56^	host^54^	Common – 1 generation/year. Overwintering as egg stage. Adult presence: May - beginning of November^42,43,52^
*Aphrodes makarovi* Zachv., 1948	*Urtica dioica*, *Taraxacum, Cirsium*^40,41,52^	not available	host^51^	Uncommon – 1 generation/year. Overwintering as egg stage. Adult presence: End of May - beginning of November^42,43,52^
*Euscelis incisus* (Kbm., 1858)	Poaceae, Fabaceae^32,40,41,52^	3 days^57^	vector^35^	Common – 3 generations/year. Overwintering as nymphal stage. Adult presence: February - mid of November^42,43,52^
*Euscelidius variegatus* (Kbm., 1858)	Poaceae^40,41,52^	3 days^57^	vector^34^	Common – 3 generations/year. Overwintering as adult stage. Adult presence: Mid of April - October^42,43,52^
*Psammotettix alienus* (Dhlb, 1850)	Poaceae^40,41,52^	not available	host^54^	Common – 2/3 generations/year. Overwintering as egg stage. Adult presence: Mid of June - beginning of October^42,43,52^
*Psammotettix confinis* (Dhlb, 1850)	Poaceae^40,41,52^	not available	—	Common – 2/3 generations/year. Overwintering as egg stage. Adult presence: Mid of June - beginning of October^42,43,52^

The eight alternative insect vectors were found largely infected by St5 CaPsol strain, prevalent in symptomatic grapevines in the examined vineyard. Even if St11 and St19 CaPsol strains were present in individuals used in transmission trials, the insects were able to vector exclusively the St5 CaPsol strain to adult grapevines. Interestingly, St5 CaPsol strain grouped in the bindweed-related *stamp* phylogenetic cluster b-II, and was extensively reported in vineyard agro-ecosystems in Italy, Austria, Germany, Macedonia, Serbia and Slovenia^9,15–17,19^. It is reasonable to hypothesize that alternative insect vectors, identified in this study, can play a role in the transmission to grapevine of at least St5 CaPsol strain in Europe. Due to their wide geographical distribution, the alternative insect vectors could be involved in CaPsol spreading also in worldwide. Furthermore, St11 and St19 CaPsol strains, grouping in the nettle-related subclusters a1 and a2, respectively, were found in 37% of the examined grapevines but in only 10% of the analyzed insects. As no insects were able to transmit such phytoplasma strains to grapevine in these experimental conditions, further investigation is necessary.

The potential significance in BN epidemiology of the insect species identified as new CaPsol vectors to grapevine is strictly related to their ecology. Considering their abundance, development cycle and adult presence in Northern Italian vineyards, known host plants, and feeding preference towards grapevine and/or common weeds in vineyards (Table 5), it is reasonable to hypothesize that *D. hamata*, *D. europaea*, *P. spumarius*, *E. incisus*, and *E. variegates* could play a role in the transmission of CaPsol to grapevine at least in the examined area. In fact, even some of them are known as oligophagous on Poaceae, in the present study these insects were found feeding on grapevine (Table 4). Moreover, as previously reported, these insects can survive on grapevine for at least two days (Table 5). Regarding *P. spumarius*, a detailed study on its feeding habit on olive revealed no indication of any puncturing of phloem tissue^37^. Nevertheless, since *P. spumarius* has been reported as a vector of the elm yellows phytoplasma^38^, in certain conditions and on certain hosts, the insect might divert its behavior from xylem to phloem feeding as some phloem feeding leafhoppers feed occasionally on xylem^39^.

Interestingly, the main host plants of *A. makarovi* are important reservoir of CaPsol; thus, also this insect could be involved in the CaPsol spreading to grapevine. Considering the lack of information about host plants and feeding preferences of *L. striatella* and *P. alienus*/*confinis*, further study are needed to investigate their potential on BN epidemiology.

The novel findings acquired in this study evidenced that the BN epidemiology in vineyard agro-ecosystems is more complex than previously known. In fact, even if tested *H. obsoletus* specimens harboured principally the bindweed-related phytoplasma strains, found prevalent within the vineyard, they were captured by netting exclusively around the borders and in the vineyard neighbourhood. For that reason, it is reasonable to hypothesize that ‘*Ca*. P. solani’ spreading to vineyard borders could be due to *H. obsoletus* and its transmission within the vineyard could be due to the feeding activity of the alternative vectors. Other studies are needed to investigate if the alternative insect vectors could be able to acquire and transmit CaPsol using the infected grapevines as source of inoculum. Furthermore, the polyvoltinism of these alternative insect vectors increases the probability of acquiring and transmitting the phytoplasma during the growing season. Accurate investigation on the ability of CaPsol acquisition related to developmental stage, the latency period, and transmission efficiency of the alternative vectors will be necessary to improve the knowledge of BN epidemiology.

The BN management, based mainly on the weeding of herbaceous plants hosting both the phytoplasma and the vectors, should consider that the eight alternative insect vectors, identified in the present study, live mainly on grasses (Table 5) and not on broad-leaves as *H. obsoletus* and *R*. *panzeri*.

## Methods

The survey was conducted in Franciacorta grape-growing area, a gently-rolling hilly zone in Lombardy Region (Northern Italy) bordering Lake Iseo. The Franciacorta is the most important Italian area for the production of sparkling wines with bottle fermentation. The grapes (more than 1,200 ha) are Chardonnay, Pinot noir, Pinot blanc and Erbamat (autoctonous variety).

The BN incidence was investigated since 2012 in 30 vineyards by symptoms observations, molecular CaPsol investigation and spatial distribution analysis of vectors and symptomatic grapevines. In one of these, the population density of *H. obsoletus* was very low within the vineyard and the adults were localized only on nettle along the borders, while few individuals of *R. panzeri* were observed in the nearby forest. The BN incidence in this vineyard was extremely high (around 30% of symptomatic grapevines in 2012 and more than 50% in 2015) with scattered distribution of symptomatic grapevines no correlated with known vector spatial distributions.

To identify alternative insect vectors, molecular investigation on Auchenorrhyncha community and symptomatic grapevines (in 2013 and 2014) and transmission trials (in 2015–2017) were conducted.

### Characteristics of target vineyard

The investigation on insect vectors of CaPsol was conducted in a 10-year old Chardonnay organic vineyard divided into two parts bordering a forest composed of broadleaf species (mainly *Castanea sativa* Mill., *Fraxinus ornus* L., *Quercus pubescens* Willd.) (N 45°35′38.12″, E 10°09′34.32″). In the both parts of the vineyard, the rows were north-south oriented and grapevines, on Kober 5BB rootstock, were trained using the Guyot system (distance between rows 2 m, plant distance along the row 0.8 m, for a total of 3876 vines). Ground cover was typical of Northern Italy with spontaneous grasses and broadleaf species. Among the agricultural practices adopted, spring weeding on the row, mowing between rows and two insecticide treatments with pyrethrum (applied at the end of June) against *Scaphoideus titanus* Ball should be mentioned because they could interfere with vineyard colonization by insects.

### Insect and grapevine samples collection

During 2013 and 2014, insects were monitored and captured every week, from May to September, by yellow sticky traps (placed within, around the borders and in the neighbourhood of the vineyard in a regular grid), sweep entomological net and pooter. The traps (21 cm × 40 cm, SuperColor Giallo®, Serbios) were positioned in the canopy of the grapevines on the support wire and on grass 0.5 m above the ground with poles. All captures were stored in ethanol 90% and identified by stereomicroscope based on phenotypic characters^40–43^. The species of genus *Psammotettix* (Dahlbom) were considered together due to difficulties in classifying the live specimens without body dissection. Based on their size and number of captures, individuals of the same taxonomic group were pooled (1–3 specimens per pool) for further molecular analyses.

Leaf petioles of symptomatic grapevine plants were also collected and stored at −30 °C. Observation of BN symptoms was made each year by the same two people. They inspected both sides of the plants in order to exclude other causes of similar symptoms (e.g. partial broken canes, *Stictocephala bisonia* Kopp and Yonke activity).

### Molecular identification and characterization of ‘*Ca*. P. solani’ in insects and grapevines

Total nucleic acids were extracted from 1150 insect pools (621 in 2013 and 529 in 2014) and leaf petioles of 60 grapevines (30 in 2013 and 2014) using the CTAB-based protocols described by Marzachì *et al*.^44^ and Angelini *et al*.^45^, respectively.

Specific detection of CaPsol was conducted by direct PCR using StampF/StampR0 primer pair followed by nested PCR with the StampF1/StampR1 primer pair, using mixtures and PCR conditions as described by Fabre *et al*.^29^. Total nucleic acids from periwinkle (*Catharanthus roseus* L. G. (Don)) plants infected by phytoplasma strains EY1 (‘*Ca*. P. ulmi’), STOL (‘*Ca*. P. solani’), and AY1 (‘*Ca*. P. asteris’) were used as reference controls. Total nucleic acids from healthy periwinkle and PCR mixture devoid of nucleic acids were used as negative controls. The presence of the nested PCR products was verified through electrophoresis on 1% agarose gel and visualized under UV transilluminator.

Fifty-eight and 54 *stamp* PCR products (StampF1/StampR1), amplified from insect specimens and grapevine samples, respectively, were sequenced in both strands (Sanger method, 5X coverage per base position) by a commercial service (Eurofins Genomics, Germany). Nucleotide sequences were assembled by the Contig Assembling Program and trimmed to the annealing sites of the nested PCR primer pair in the software BioEdit, version 7.2.6^46^.

Nucleotide sequences of *stamp* gene, amplified from the CaPsol strains detected in the examined grapevine samples and insect specimens, were aligned using the ClustalW Multiple Alignment program in the software BioEdit and analysed by Sequence Identity Matrix to estimate their genetic diversity. *Stamp* sequence variants, identified in the study, were aligned and compared with representative sequences of previously defined sequence variants^19^; a nucleotide sequence identity of 100% was necessary for the attribution to such sequence variants.

### ‘*Ca*. P. solani’ phylogenetic analysis

*Stamp* gene nucleotide sequences of CaPsol representative strains of GuSt (*stamp*) sequence variants, identified in this and in previous studies^19^, were aligned and used for generating unrooted phylogenetic trees by Neighbor-Joining method performed using the Jukes-Cantor model and bootstrap replicated 1000 times in the MEGA6 software^47^. All positions with less than 95.0% site coverage were eliminated. That is, fewer than 5% alignment gaps, missing data, and ambiguous bases were allowed at any position.

### Transmission trials

Based on the results obtained from Auchenorrhyncha community description and molecular analysis of CaPsol strains, insects to be tested in transmission trials for their CaPsol vectoring activity to grapevine were selected using the following three criteria simultaneously satisfied in at least 2013 and/or 2014: (i) high abundance (>25 individuals collected in the vineyard over the vegetative season); (ii) high infection rate (>10% of CaPsol-infected insect pools); (iii) the harbouring of CaPsol strain characterized by *stamp* sequence variant found also in grapevine.

A total of 550 individuals of the 10 selected insect species were captured in the examined vineyard on three sampling days in 2015 (June 11 and 25; July 7) and five days in 2016 (June 28; July 6 and 21; August 3 and 28). The specimens of each insect species were captured, on each sampling date, using sweep net and pooter on the canopy of symptomatic grapevines and on the grassing near them. The captured insects were kept in jars for transport to the laboratory for their classification. Collected insects were kept alive and in the conditions required for survival and good fitness maintenance. After the classification, the insects were forced to feed on asymptomatic and PCR-tested phytoplasma-free potted grapevines (cv. Chardonnay) previously treated with hot water. Forty-four transmission trials were conducted in a greenhouse under controlled conditions [25 ± 3 °C, 70 ± 5 RH 16:8 (L:D) daily light cycle], located in Verona province (45°20′13.72″N; 11°13′03.28″E), and left till the end of adult survival. After this period, the plants were kept in an insect-free greenhouse in both years. On each date, one phytoplasma-free grapevine plant was maintained without insects as control. Dead insects were stored at −30 °C. CaPsol was detected by nested PCR-based amplification of *stamp* gene^29^ and by SYBR Green real-time amplification assay^48^ using as templates the total nucleic acids extracted from both the individual insect specimens and the petioles of grapevine plants collected in October 2015 and July 2016 for the trials performed in 2015, and in October 2016 and July 2017 for the trials performed in 2016. In real-time amplification assay, only amplified PCR products showing a Tm of 81.5 ± 0.2 °C and a Ct < 37 were associated with the presence of CaPsol, as previously described^20^. CaPsol strains detected in insects and plants were characterized through nucleotide sequence analyses of *stamp* amplicons as described above.

### Plants eaten by the insects

In order to identify the plant species on which the insects have had their last meal, a molecular characterization of the gut contents has been performed for 154 insect specimens captured in the examined vineyard in 2016 simultaneously to those captured for transmission trials. A fragment (750 bp) of *rbcL* gene, coding for the plant plastid ribulose-bisphosphate carboxylase large subunit, was amplified by PCR using primers rbcL1F/rbcL724R as previously described^49,50^. Nucleotide sequences of *rbcL* gene, amplified from insect specimens, were sequenced in both strands (Sanger method, 5X coverage per base position) by a commercial service (Eurofins Genomics). Nucleotide sequences were assembled by the Contig Assembling Program, trimmed to the annealing sites of primer pair in the software BioEdit, compared to GeneBank through BLAST analysis (http://www.ncbi.nlm.nih.gov/blast).

## Supplementary information

Figure S1

## Data Availability

All data generated or analysed during this study are included in this published article (and its Supplementary Information files).
