# Supplementary material for: Identification and ecology of alternative insect vectors of ‘Candidatus Phytoplasma solani’ to grapevine
Source: Sci Rep. 2019 Dec 20;9:19522. doi: 10.1038/s41598-019-56076-9 (PMC6925216; doi:10.1038/s41598-019-56076-9)

**Identification and ecology of alternative insect vectors of '*Candidatus* Phytoplasma solani’ to grapevine**

Fabio Quaglino^1^, Francesco Sanna^2^, Abdelhameed Moussa^1^, Monica Faccincani^3^, Alessandro Passera^1^, Paola Casati^1^, Piero Attilio Bianco^1^ & Nicola Mori^2*^

^1^Dipartimento di Scienze Agrarie e Ambientali - Produzione, Territorio, Agroenergia, Università degli Studi di Milano, via Celoria 2, 20133 Milano;

^2^Dipartimento di Agronomia Animali Alimenti Risorse Naturali e Ambiente, Università degli Studi di Padova, Agripolis - viale dell'università, 16 - Legnaro (Padova);

^3^Consorzio per la tutela del Franciacorta - via G. Verdi 53, 25030 Erbusco (BS)

^*^**Corresponding author**: Nicola Mori; e-mail: nicola.mori@unipd.it; phone: +39-049-8272802

**Supplementary Information**

**Figure S1.** *Stamp* sequence variants in BNp hosts grouped in phylogenetic clusters


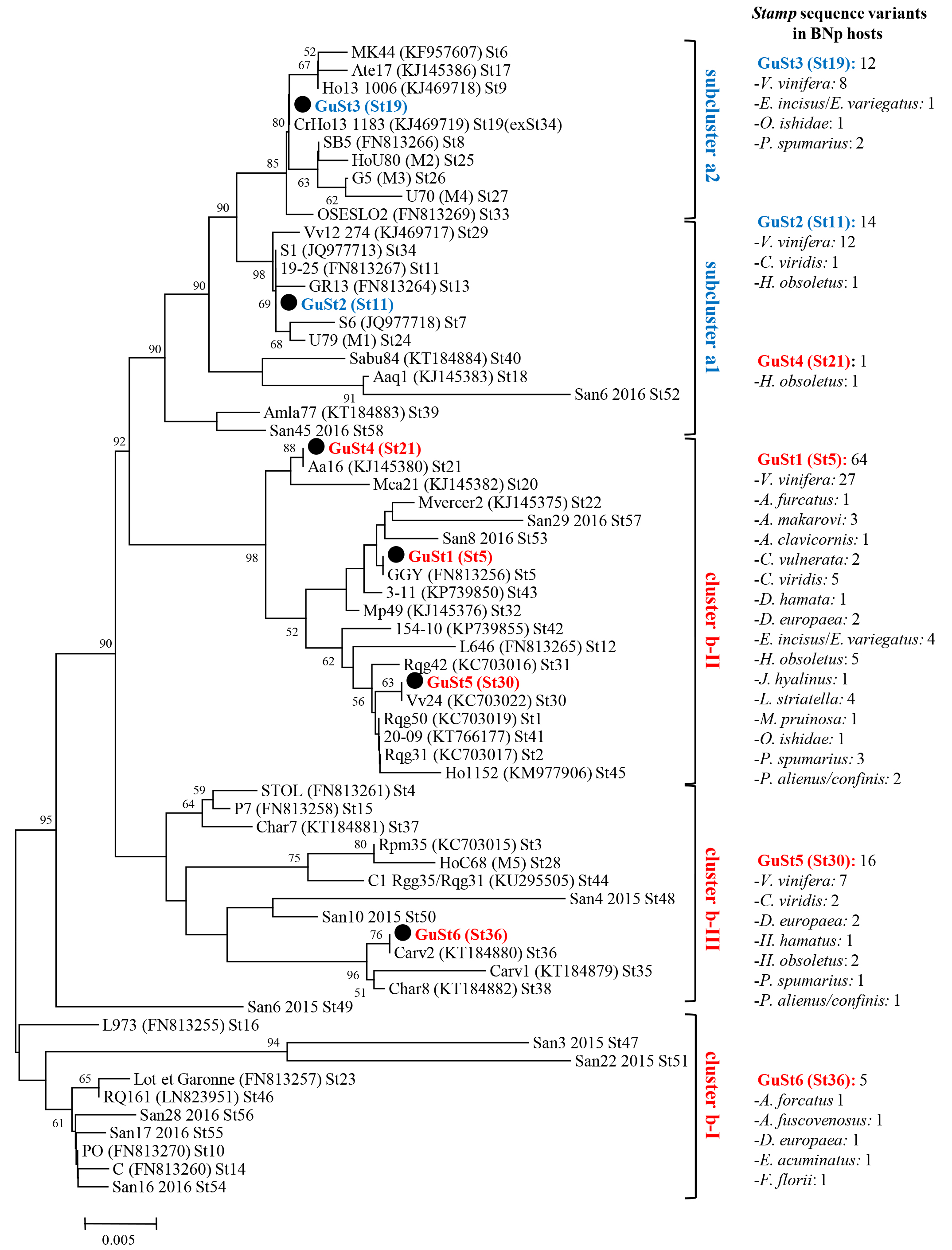

Supplement: Supplementary file 1 — Figure S1 [file 41598_2019_56076_MOESM1_ESM.doc]
